# Interactions between folate intake and genetic predictors of gene expression levels associated with colorectal cancer risk

**DOI:** 10.1038/s41598-022-23451-y

**Published:** 2022-11-07

**Authors:** Cameron B. Haas, Yu-Ru Su, Paneen Petersen, Xiaoliang Wang, Stephanie A. Bien, Yi Lin, Demetrius Albanes, Stephanie J. Weinstein, Mark A. Jenkins, Jane C. Figueiredo, Polly A. Newcomb, Graham Casey, Loic Le Marchand, Peter T. Campbell, Victor Moreno, John D. Potter, Lori C. Sakoda, Martha L. Slattery, Andrew T. Chan, Li Li, Graham G. Giles, Roger L. Milne, Stephen B. Gruber, Gad Rennert, Michael O. Woods, Steven J. Gallinger, Sonja Berndt, Richard B. Hayes, Wen-Yi Huang, Alicja Wolk, Emily White, Hongmei Nan, Rami Nassir, Noralane M. Lindor, Juan P. Lewinger, Andre E. Kim, David Conti, W. James Gauderman, Daniel D. Buchanan, Ulrike Peters, Li Hsu

**Affiliations:** 1 Department of Epidemiology, University of Washington, Seattle, WA USA; 2 Public Health Sciences Division, Fred Hutchinson Cancer Center, Seattle, WA USA; 3 Division of Cancer Epidemiology and Genetics, National Cancer Institute, National Institutes of Health, Bethesda, MD USA; 4 Centre for Epidemiology and Biostatistics, Melbourne School of Population and Global Health, The University of Melbourne, Melbourne, VIC Australia; 5 Department of Medicine, Samuel Oschin Comprehensive Cancer Institute, Cedars-Sinai Medical Center, Los Angeles, CA USA; 6 Department of Preventive Medicine and USC Norris Comprehensive Cancer Center, Keck School of Medicine, University of Southern California, Los Angeles, CA USA; 7 School of Public Health, University of Washington, Seattle, WA USA; 8 Center for Public Health Genomics, University of Virginia, Charlottesville, VA USA; 9 University of Hawaii Cancer Center, Honolulu, HI USA; 10 Behavioral and Epidemiology Research Group, American Cancer Society, Atlanta, GA USA; 11 Oncology Data Analytics Program, Catalan Institute of Oncology-IDIBELL, L’Hospitalet de Llobregat, Barcelona, Spain; 12 CIBER Epidemiología y Salud Pública (CIBERESP), Madrid, Spain; 13 Department of Clinical Sciences, Faculty of Medicine, University of Barcelona, Barcelona, Spain; 14 ONCOBEL Program, Bellvitge Biomedical Research Institute (IDIBELL), L’Hospitalet de Llobregat, Barcelona, Spain; 15 Center for Public Health Research, Massey University, Wellington, New Zealand; 16 Division of Research, Kaiser Permanente Northern California, Oakland, CA USA; 17 Department of Internal Medicine, University of Utah, Salt Lake City, UT USA; 18 Division of Gastroenterology, Massachusetts General Hospital and Harvard Medical School, Boston, MA USA; 19 Channing Division of Network Medicine, Brigham and Women’s Hospital and Harvard Medical School, Boston, MA USA; 20 Clinical and Translational Epidemiology Unit, Massachusetts General Hospital and Harvard Medical School, Boston, MA USA; 21 Broad Institute of Harvard and MIT, Cambridge, MA USA; 22 Department of Family Medicine, University of Virginia, Charlottesville, VA USA; 23 Cancer Epidemiology Division, Cancer Council Victoria, Melbourne, VIC Australia; 24 Precision Medicine, School of Clinical Sciences at Monash Health, Monash University, Clayton, VIC Australia; 25 Department of Community Medicine and Epidemiology, Lady Davis Carmel Medical Center, Haifa, Israel; 26 Ruth and Bruce Rappaport Faculty of Medicine, Technion-Israel Institute of Technology, Haifa, Israel; 27 Clalit National Cancer Control Center, Haifa, Israel; 28 Memorial University of Newfoundland, Discipline of Genetics, St. John’s, Canada; 29 Lunenfeld Tanenbaum Research Institute, Mount Sinai Hospital, University of Toronto, Toronto, ON Canada; 30 Division of Epidemiology, Department of Population Health, New York University School of Medicine, New York, NY USA; 31 Institute of Environmental Medicine, Karolinska Institutet, Stockholm, Sweden; 32 IU Melvin and Bren Simon Cancer Center, Indiana University, Indianapolis, IN USA; 33 Department of Pathology, School of Medicine, Umm Al-Qura’a University, Makkah, Saudi Arabia; 34 Department of Health Science Research, Mayo Clinic, Scottsdale, AZ USA; 35 Department of Preventive Medicine, Keck School of Medicine, University of Southern California, Los Angeles, CA USA; 36 Colorectal Oncogenomics Group, Department of Clinical Pathology, The University of Melbourne, Parkville, VIC 3010 Australia; 37 University of Melbourne Centre for Cancer Research, Victorian Comprehensive Cancer Centre, Parkville, VIC 3010 Australia; 38 Department of Biostatistics, University of Washington, Seattle, WA USA

**Keywords:** Cancer genetics, Cancer genomics, Functional genomics, Gene expression, Genetic association study, Genetic interaction, Cancer, Computational biology and bioinformatics, Genetics, Biomarkers, Risk factors

## Abstract

Observational studies have shown higher folate consumption to be associated with lower risk of colorectal cancer (CRC). Understanding whether and how genetic risk factors interact with folate could further elucidate the underlying mechanism. Aggregating functionally relevant genetic variants in set-based variant testing has higher power to detect gene–environment (G × E) interactions and may provide information on the underlying biological pathway. We investigated interactions between folate consumption and predicted gene expression on colorectal cancer risk across the genome. We used variant weights from the PrediXcan models of colon tissue-specific gene expression as a priori variant information for a set-based G × E approach. We harmonized total folate intake (mcg/day) based on dietary intake and supplemental use across cohort and case–control studies and calculated sex and study specific quantiles. Analyses were performed using a mixed effects score tests for interactions between folate and genetically predicted expression of 4839 genes with available genetically predicted expression. We pooled results across 23 studies for a total of 13,498 cases with colorectal tumors and 13,918 controls of European ancestry. We used a false discovery rate of 0.2 to identify genes with suggestive evidence of an interaction. We found suggestive evidence of interaction with folate intake on CRC risk for genes including glutathione S-Transferase Alpha 1 (*GSTA1*; p = 4.3E−4), Tonsuko Like, DNA Repair Protein (*TONSL*; p = 4.3E−4), and Aspartylglucosaminidase (*AGA*: p = 4.5E−4). We identified three genes involved in preventing or repairing DNA damage that may interact with folate consumption to alter CRC risk. Glutathione is an antioxidant, preventing cellular damage and is a downstream metabolite of homocysteine and metabolized by *GSTA1*. *TONSL* is part of a complex that functions in the recovery of double strand breaks and *AGA* plays a role in lysosomal breakdown of glycoprotein.

## Introduction

Folate is a naturally occurring, water-soluble B vitamin that cannot be produced by the human body and plays a key role in DNA formation and is necessary for cellular division and tissue differentiation. It is found abundantly in green leafy vegetables, legumes, fruits, and its more potent form, folic acid, is found in supplements and fortified foods^1^. Supplementary folic acid is routinely prescribed during pregnancy as an evidence-based intervention to prevent neural tube defects in utero^2,3^. Dietary deficiency is typically found in persons subsisting on inadequate diets, as well as chronic alcoholics with diminished absorption^4^. Fortification of grains with folic acid began in the early 1990s to prevent nutritional deficiencies^5,6^. To date, 71 countries have legislative mandates for including folate in the fortification of milled grains^5^. Results pre- and post-fortification and risk of CRC have been somewhat inconsistent^7–13^, suggesting that folate might play a more complex role in colorectal carcinogenesis through various interactions^14–16^. Given the complexity of the relationship between CRC and folate, there is a need to elucidate the underlying biological mechanisms and possible differential risk based on individual genetics^15^.

Increased folic acid consumption is known to lower circulating levels of homocysteine, a common amino acid that has been associated with numerous diseases^6,17,18^. The absence of folic acid leads to impaired DNA synthesis and disturbances in red blood cell maturation^19^. Due to its role as a carrier of one-carbon groups and in folate-mediated one-carbon metabolism (FOCM), insufficient folate consumption has been implicated as a possible cause of cancer^12,20–23^. Consistent with this hypothesis previous studies have shown evidence that greater folate intake is associated with a reduced risk of colorectal adenomas and cancers (CRC)^11,21,24^. A pooled analysis of 13 prospective studies in 2010 observed a modest effect, estimating a 2% risk reduction for CRC per 100 μg/day increase in total folate consumption^25^.

Candidate gene approaches targeting FOCM-related genes have shown associations with CRC risk^24,26,27^. This has raised interest in studying interactions between folate and genetic variants^23,28^. As such, it has been hypothesized that germline mutations to the enzyme 5,10-methylenetetrahydrofolate reductase (MTHFR) would be a driver of the effects on folate on CRC risk^11,29,30^. A common mutation, 677TT in MTHFR has been associated with a greater decreased risk of CRC in high consumers of folate and low alcohol consumption^27,29,31,32^ compared to lower folate consumers. However, such analyses have relied on the assumption that FOCM-related genes are the driving genetic force on the pathway from folate consumption to CRC development. A genome-wide approach has the potential to identify novel genes that may modify the folate–CRC association.

To this end, we conducted a novel set-based genome-wide analysis to test interactions between genes and total folate intake on CRC risk. By using a set-based approach we may increase the power to detect associations, which is a common issue in traditional gene–environment interaction studies. We incorporate functional annotation based weights from PrediXcan, a transcriptome prediction tool^33^.

## Methods

### Study participants

We used epidemiological and genetic data from studies included in three international CRC consortia: the Genetics and Epidemiology of Colorectal Cancer Consortium (GECCO), the Colorectal Transdisciplinary Study (CORECT) and the Colon Cancer Family Registry (CCFR). Full details have been published previously^34,35^, and the demographic characteristics of study participants are summarized in Table 1. We describe the study designs in Supplementary Table 1A and present results for the study design specific effects of total folate on CRC for study designs in Supplementary Table 1B. In case–control study designs, included cases were ascertained using population-based sampling and age-matched controls. In prospective cohorts, cases were identified through linkage to cancer registries. Participants with non-European ancestry were excluded due to small sample sizes among those with genetic data. Informed consent was given by all participants, and studies were approved by their respective Institutional Review Boards and complies with all relevant ethical regulations.

**Table 1:** Characteristics of participants from all studies by colorectal cancer case/control status and Chi-square p-values for statistical differences.

	Cases	Controls	p-value
N	13,498	13,918	
Male (%)	6190 (45.9%)	6014 (43.2%)	< 0.001*
Mean reference age (SD)	65.0 (10.5)	65.0 (9.9)	0.971
**BMI in kg/m**^**2**^** (%)**			
Normal (18.5–24.9)	4415 (34.0%)	5260 (39.0%)	< 0.001*
Overweight (25–30)	5355 (41.2%)	5451 (40.4%)	
Obese (≥ 30)	3215 (24.8%)	2782 (20.6%)	
Mean total energy consumption in kcal/day (SD)	1938.3 (769.1)	1911.3 (718.7)	0.003*
**Sex-study specific quantile of total folate consumption in mcg/day (%)**			
First quantile	3399 (25.2%)	3176 (22.8%)	< 0.001*
Second quantile	3089 (22.9%)	3235 (23.2%)	
Third quantile	4120 (30.5%)	4162 (29.9%)	
Fourth quantile	2890 (21.4%)	3345 (24.0%)	
**Alcohol consumption in g/day (%)**			< 0.001*
Nondrinker	7031 (56.2%)	6852 (52.6%)	
1–28 g/day	4464 (35.7%)	5250 (40.3%)	
> 28 g/day	1017 (8.1%)	924 (7.1%)	
**Smoking status**			0.626
Never smoker	6452 (48.6%)	6736 (49.4%)	
Former smoker	5271 (39.8%)	5328 (39.1%)	
Smoker	1530 (11.5%)	1560 (11.5%)	

Continuous variables were presented as mean and standard deviation (SD) and p-values were calculated using Student’s *t* test for difference in means; categorical variables were presented as n (%) and p-values were calculated using Pearson Chi-square test.

*Statistically significant difference between cases and controls.

### Genotype data

Details on genotyping and imputation have been reported previously^36^. In brief, DNA was mostly obtained from blood samples, with some from buccal swabs. Several platforms (the Illumina HumanHap 300k, 240k, 550k and OncoArray 610k BeadChip Array system, or Affymetrix platform) were used for genotyping^37,38^. Samples were excluded on the basis of sample call rate ≤ 97%, heterozygosity, unexpected duplicates or relative pairs, gender discrepancy and principal component analysis (PCA) outlier of HapMap2 CEU cluster. SNPs were excluded on the basis of inconsistency across platforms, call rate < 98%, and out of Hardy–Weinberg equilibrium (HWE) in controls (p < 0.0001)^37^. SNPs were imputed to the CEU population in Haplotype Reference Consortium (HRC version r1.0) if not directly genotyped^39^, and restricted by imputation accuracy (R^2^ > 0.3).

### Genetically predicted gene expression

The sets of genetic variants and weights for predicting gene expression were downloaded from the publicly available PredictDB Repository (https://hakyimlab.org/resource/predixcan/). The weights for the predicted gene expression were obtained by an elastic net penalized regression approach using the genome-wide variant data and transcriptome data from 169 colon tissue samples from the GTEx project (GTEx v6)^40^ (Supplementary material). We restricted GTEx data to the transverse colon as it included the entire colonic wall and as such the epithelial layer in the mucosa most relevant to CC development while the GTEx sigmoid colon data only included the muscle layer. Genes for which SNPs explained at least 1% of the variation in CRC risk were selected for interaction analyses. A total of 4839 genes were included.

### Exposure assessment

Basic demographics and environmental risk factors were collected using in-person interviews and/or structured questionnaires^35,41–49^. For these data, we carried out a multi-step data harmonization procedure, reconciling each study’s unique protocols and data-collection instruments as discussed previously^34^. Folate and folic acid intake were assessed at the reference time using food frequency questionnaires (FFQs). For cohort studies, the reference time was time of enrollment or blood collection. Folate and folic acid intake in each study were determined based on micrograms per day (mcg/day) of folate from foods (i.e., dietary folate) and mcg/day of folic acid from supplements (single or multivitamins) when available. Only two of the 23 studies with dietary folate intake did not capture information regarding supplemental folate. To account for the higher bioavailability of synthetic folic acid vs. natural folate in foods, we calculated total folate intake as dietary folate equivalents (total mcg DFE = mcg of dietary folate + 1.7 × mcg folic acid from supplements)^50^. Because the time of enrollment for some studies overlapped or followed the period of folic acid fortification (1996–1998), these studies accounted for folic acid fortification when calculating dietary folate intake and entered dietary folate intake as mcg of natural food folate + 1.7 × mcg folic acid from fortified food (see Supplementary Table 1A). Two studies (OFCCR, DALS) entered supplement data as regular user vs. nonuser; for these, we assumed regular use was 400 mcg/day or 400 mcg/tablet (for multivitamins), which corresponds to the generic dose in supplements^25,51^. The primary analysis used sex-study specific quartiles of total folate using controls based on the calculated daily dietary and supplemental intake, if available. By using categorical sex-study specific quartiles we reduce the influence of outliers and skewed distributions and is consistent with the Cancer Cohort Pooling Project^52^. To further explore the differences in bioavailability, secondary analyses we explored sex-study specific quartiles of dietary folate and binary (yes/no) supplemental folate separately.

### Statistical analysis

We used the Mixed effects Score Tests for interaction (MiSTi)^53^, a mixed effects score test for gene-based interaction test with folate consumption on CRC risk, to conduct a pooled analysis across all studies. MiSTi modeled the gene–environmental interaction effect by two components. The fixed effects component incorporates variant functional information from PrediXcan as weights with our genotype data to calculate the genetically predicted gene expression and then assess its interaction with folate consumption. The random effects component involves residual interaction effects that have not been accounted for by the fixed effects. We used sex- and study-specific quantiles of folate consumption. p-values were calculated separately for fixed and random effects interaction terms, after adjusting for age, sex, study, sex-study specific quartiles of total energy consumption in kcal, and principal components to account for population stratification. We used the MiSTi data-adaptive weighted combination approach to combine the fixed and random effects components.

Genes with p-values less than the Bonferroni correction (0.05/4839 = 1.03E−5) were considered genome-wide statistically significant for an interaction with folate. p-values that reached false discovery rate (FDR) at 20% were considered having suggestive evidence of interaction as it is less stringent than a Bonferroni threshold. We conducted follow-up analyses based on the fixed and random effects p-values. For associations driven by the fixed effects, we investigated the direction and magnitude of these interactions using the generalized linear model, which included all covariates in the original model, folate, standardized predicted gene expression, and an interaction term for folate and predicted gene expression. Genes for which the signal was driven by the random effects component were further investigated to identify individual variants of the gene set as drivers using the same approach with interactions for individual variants and folate while adjusting for all other variants in the gene set. Due to some of the variants having high collinearity, we pruned variants by R^2^ < 0.9.

All analyses were performed using R version 4.0.1^54^.

We performed these additional follow-up analyses for MTHFR, as prior candidate gene studies have shown variants, specifically the C677T mutation, alter the association between folate and CRC^31,32,55–58^. We additionally include the results of the gene–environment interaction between rs1801133 (C677T mutation) per additional effect allele with sex-study specific quantiles of total folate consumption on colorectal cancer.

## Results

The final sample included 13,498 cases and 13,918 controls with both folate and energy consumption measures available from 23 studies. We present demographic characteristics of all samples and report on measures for factors associated with CRC risk for study participants by case–control status in Table 1. Cases were more likely to be male, have higher BMI, and report consuming less folate daily and more calories daily compared to controls. Multivariable logistic regression estimated a reduced risk of CRC per-quartile increase in total folate intake, adjusting for sex, age at reference, and total energy intake, and study (OR = 0.91, 95% CI: 0.89, 0.93, p-trend < 0.001, Supplementary Table 1B). Sensitivity analyses included further adjustment for smoking and alcohol consumption, which had little effect on the estimates for total folate and CRC risk.

We found no suggestion of interaction between predicted gene expression for the *MTHFR* gene and sex-study specific folate on risk of CRC in our analysis. Supplementary Table 2A–C present follow-up analyses conducted to test the interaction per standard deviation change in predicted gene expression within sex-study specific quantiles, allowing for a non-linear relationship between folate quantiles, as well as individual variant weights used in the modeling of predicted gene expression to capture the C677T mutation. In the snp-environment interaction analysis for the rs1801133 variant (C677T mutation), no interaction was show between each additional effect allele with sex-study specific quantiles of total folate consumption on risk of CRC (ratio of odds ratio = 1.02; 95% CI = 0.98, 1.06; interaction p-value = 0.235).

**Figure 1:**
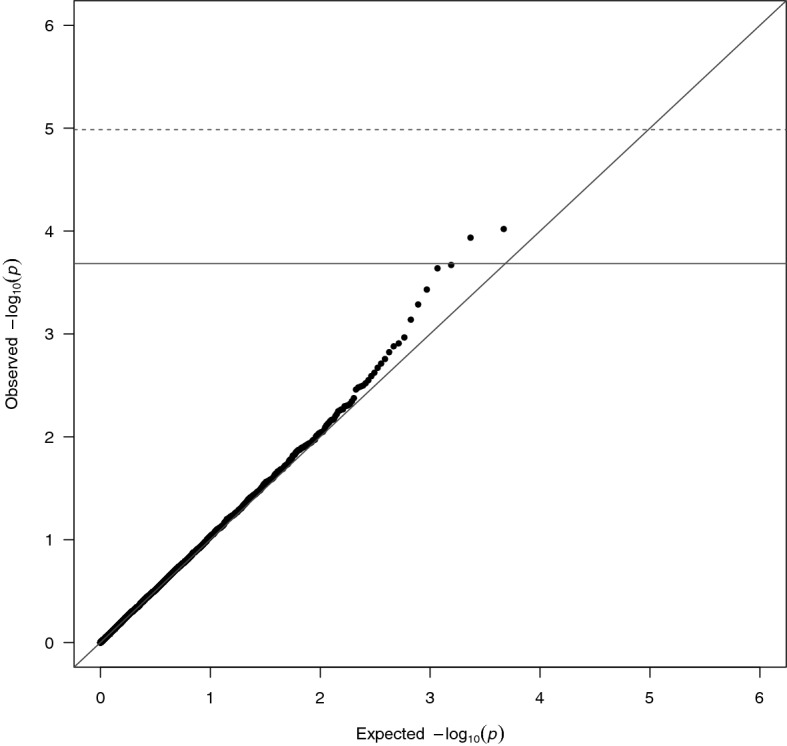
MiSTi results for Adaptive Weight test of predicted gene expression interactions with total folate intake. Dashed line is the Bonferroni corrected threshold, solid is the false discovery rate < 0.2 threshold for p-value significance.

The median number of SNPs included in the gene sets was 25 (minimum: 1, inter-quartile range [IQR]: 13–43, maximum: 277). Figure 1 displays the quantile–quantile plot for the G × E test that combined both fixed and random effects using adaptive weight. While there was no G × E interaction that reached the Bonferroni threshold (0.05/4839), three did surpass the false discover rate (FDR) of 0.2.

We present the findings with p-values that surpassed the FDR threshold for gene interactions with total folate consumption and CRC risk in Table 2. We observed suggestive evidence of interactions between total folate intake and 3 independent gene sets on risk of CRC at FDR < 0.2, including Glutathione S-Transferase Alpha 1 (*GSTA1*; p = 4.3E−4), Tonsuko Like, DNA Repair Protein (*TONSL*; p = 4.3E−4), and Aspartylglucosaminidase (*AGA*; p = 4.5E−4). In follow-up analyses for these three genes we observed positive interactions for *GST1A* and *AGA*, showing greater risk for CRC associated with higher gene expression and increasing folate consumption (Table 3). As the signal for *TONSL* primarily came from the random effects, indicating one or a few variants were drivers of the association, we investigated the individual interactions of variants with sex-study specific folate. We see two variants as possible drivers of the signal in our main analysis, 8:144964455_T/C and 8:144965104, as shown in Table 4.

**Table 2:** Top genes from MiSTi for gene-based interactions with total folate for colorectal cancer based on Adaptive Weight Test p-values with FDR < 0.2.

Gene	Chromosome	R^2^	Number of SNPs	Fixed effects	Random effects	Adaptive weight P
*GSTA1*	6	0.17	20	1.6E−4	0.11	4.3E−4
*TONSL*	8	0.06	28	0.048	1.3E−3	4.3E−4
*AGA*	4	0.37	39	1.2E−4	0.65	4.5E−4

Models were adjusted for age, sex, study, sex-study specific quartiles of total energy consumption in kcal, and principal components to account for population stratification.

**Table 3:** Estimated odds ratios (ORs) and 95% confidence intervals (CIs) of colorectal cancer risk per standard deviation change in predicted gene expression stratified by sex-study specific quantiles of total folate consumption.

Gene	Sex-study specific quantile of total folate	OR	95% CI	Interaction p-value*
*GSTA1*	1	1.29	0.86, 1.92	
	2	1.32	0.88, 1.98	0.48
	3	1.40	0.93, 2.10	0.01
	4	1.45	0.97, 2.17	6.5E−4
*AGA*	1	1.06	0.79, 1.43	
	2	1.16	0.85, 1.58	0.01
	3	1.15	0.85, 1.56	0.02
	4	1.21	0.89, 1.64	3.6E−4

Models were adjusted for age, sex, study, sex-study specific quartiles of total energy consumption in kcal, and principal components to account for population stratification.

*p-value tests for difference in quantile specific OR estimate for per standard deviation change in predicted gene expression and the lowest sex-study specific quantile of total folate quantile on colorectal cancer risk.

**Table 4:** Variant specific ratio of odds ratios associated with per quantile increase in sex-study specific quantiles of total folate consumption on colorectal cancer risk for variants included in TONSL*.

Variant**	Ratio of odds ratio	Standard error	Interaction p-value
8:144757296_A/G	1.04	0.22	0.86
8:144801243_C/T	1.03	0.02	0.09
8:144801593_C/A	0.93	0.04	0.10
8:144856443_G/T	0.82	0.13	0.13
8:144964455_T/C	0.95	0.02	7.0E−3
8:144965104_G/A	0.95	0.02	5.1E−3
8:145651888_G/A	1.00	0.02	0.78
8:145653006_G/A	1.00	0.02	0.76
8:145668042_G/A	1.01	0.02	0.76
8:146216936_A/G	0.96	0.02	0.03
8:146280802_C/T	1.03	0.02	0.19

Models were adjusted for age, sex, study, sex-study specific quartiles of total energy consumption in kcal, and principal components to account for population stratification.

*Filtering to uncorrelated variants in TONSL (r^2^ < 0.9).

**Chromosomal position and reference/alternative alleles.

## Discussion

In this sizable analysis including a large number of studies we harmonized data on folate consumption and genome-wide genetic data to investigate interactions between folate intake and variants in genes on CRC risk. We observed an inverse association between folate intake and CRC risk across 23 studies. Using our novel statistical set-based G × E mixed effects score tests, MiSTi, we identified 3 genes with suggestive interactive effects with total folate consumption on CRC risk: *GSTA1*, *TONSL*, and *AGA*.

We observed a positive interaction between the predicted gene expression of *GSTA1* and folate for CRC risk. *GSTA1* located at 6p12.2 encodes for an enzyme that functions in cellular detoxification of electrophilic compounds through glutathione metabolism. Electrophilic compounds include carcinogens, therapeutic drugs, environmental toxins, and products of oxidative stress. Glutathione is a product of homocysteine metabolism, a key amino acid correlated with folate intake, and is bound to free radicals by *GSTA1*^59^. Our results suggest that folate consumption may increase remethylation of homocysteine to methionine, thus reducing the production of glutathione need for DNA repair. Mutations in *GSTA1* could feasibly alter the binding affinity of glutathione to carcinogenic compounds, leading to variation in cancer susceptibility. Of the 20 SNPs included in our analyses of *GSTA1*, three of the alternative alleles result in missense mutations to the gene^60^. Compromised function of glutathione as an antioxidant due to mutations in *GSTA1* in conjunction with depleted levels of glutathione due to lower homocysteine levels may be a pathway to tumorigenesis^6,22^. Candidate gene studies have shown no association between *GSTA1* and colorectal cancer or adenoma risk^61,62^. However, previous studies have shown interactions between diet, such as cruciferous vegetable consumption, and *GSTA1* genotypes, supporting that associations between this gene and CRC are likely driven by dietary exposures^63–65^.

*TONSL* in the 8q24.3 region codes for a 1378 amino acid protein component of the MMS22L-TONSL complex, which functions in recovery of damaged replication forks^66^. Numerous mutations in *TONSL* are considered pathogenic^60^. Low levels of the MMS22L-TONSL complex result in increased frequency of DNA double-strand breaks and compromised DNA integrity^66^. In combination with increased DNA damage due to deficiencies in folic acid, impaired functionality of the MMS22L-TONSL due to functional mutations may be a pathway to increase tumorigenesis. Follow-up analyses further suggested that possible associations may be primarily driven by a small subset of variants included in our gene set in the main analysis.

We observed increasing risk of CRC per standard deviation increase in predicted gene expression of *AGA* with increasing folate consumption. The *AGA* gene is in the 4q34.3 region and codes for a 346 amino acid protein that functions in pathways related to the innate immune system and asparagine degradation^67^. Once the protein is processed into the mature enzyme it takes part in the catabolism of N-linked oligosaccharides, cleaving asparagine from *N*-acetylglucosamines in one of the final steps in the lysosomal breakdown of glycoproteins. Mutations in the *AGA* gene are known to cause the lysosomal storage disease aspartylglycosaminuria, eventually resulting in neurodegeneration. Previous research has not indicated a link to cancer for this gene.

While we have many strengths in performing the largest investigation of gene–folate interactions to date using a powerful set-based approach that allows to account for functional prediction, some limitations should be considered when interpreting these findings. Approximately half of the studies in our consortium ascertained cases using a cohort study design which may have resulted in earlier and more frequent detection of tumors. Most cohort studies in our consortium used population-based registries for case ascertain. However, one study, The Prostate, Lung, Colorectal, and Ovarian Cancer Screening Trial, was a randomized trial to determine the effectiveness of screening. While we have adjusted for study in our approach there may be unknown residual effects of this design. Our study population was limited to those of European descent. As gene expression levels may differ across populations of different ancestry, our results may not be generalizable to populations of non-European ancestry. The studies included in our analysis occurred over a range of time and geographic locations. Fortification with folate occurred in different places at different times and we used adjusted dietary equivalents to account for these differences (see Supplementary Table 1A). Study designs also varied. We looked at the effect size of folate on CRC by case/control versus cohort study designs and did not find a substantive difference to justify stratified analyses (see Supplementary Table 1B). Lastly, studies in our consortium generally ascertained folate consumption through standard questionnaires. However, previous work has shown self-reported measures of folate intake to be positively and moderately correlated with plasma levels of folate, particularly when dietary supplement use was included as was generally the case in studies included in our analyses^68^.

We utilized colon-specific gene expression data, specifically transverse colon tissue captured by the GTex Project^40^. One limitation of this data is the diversity of cell types aside from epithelial cells of the mucosa of the colon, from which CRC derives given that the entire colonic wall was sampled. The impact of this would cause a dilution of gene expression for the tissue most relevant for CRC. However, we expect this to be an improvement over alternative tissue types including blood or sigmoid colon tissues in GTEx, which were collected from muscle tissues only and would not represent the gene expression profile of interest.

Although MiSTi is a powerful statistical tool, which accounts for both fixed- and random-effects of the gene–folate interaction, none of our findings reached the Bonferroni corrected threshold, which can be overly conservative as many genes are co-expressed. We did not perform independent replication and thus follow-up investigations are warranted, as a FDR of 0.2 should be considered liberal^53^. The previously suggested *MTHFR* gene was not identified in our analysis^27^. However, in using the penalized elastic net to create our predicted gene expression the C677T was not included in the variant weights due to the insignificant contribution to regulation of gene expression. While it was also not seen in the gene–environment interaction analysis either, we believe these results to be representative of an agnostic approach which has not been shown before, as opposed to candidate gene studies.

Our analysis was conducted in the largest pooled analysis of a well characterized and harmonized consortium of CRC with comprehensive genetic data which enabled a hypothesis-free genome-wide investigation of interactions with folate consumption on CRC risk. An extensive number of genes evaluated in prior candidate gene–folate interaction studies, including *MTHFR*, were included among the 4839 genes examined. However, none of those previously hypothesized genes were found to interact with folate consumption in our analysis^31,57,69^. We conducted additional follow-up analysis for *MTHFR* using indicator terms for sex-study-specific folate quantiles and interaction terms for all quantiles with predicted gene expression were null (see Supplementary Table 2A–C). No previous study has agnostically tested for genetic interactions with folate for cancer. Our statistical approach was potentially improved by incorporating functional variant weights and testing gene-sets rather than individual SNPs reducing the penalty for multiple testing. In the end, we found three genes that were suggestive of interacting with folate consumption on risk of CRC, supporting the hypothesis that associations of folate with CRC may be modified by common genetic variation.

The biological functions of our top genes serve to primarily prevent or repair DNA damage. The combined effects of increased DNA damage due to folate deficiencies and compromised functionality of these genes may be an important pathway in CRC tumorigenesis. These findings, particularly for *GSTA1*, warrant follow-up in future studies with comprehensive genetic and data on folate intake in order to confirm the potential role of these genes in interacting with folate on CRC risk.

## Supplementary Information

Supplementary Information 1.

Supplementary Information 2.

## Data Availability

Data will be made available upon request and approval by contacting Dr. Ulrike Peters.

## Code availability

Please contact the corresponding author for code.
