# Supplementary material for: Interactions between folate intake and genetic predictors of gene expression levels associated with colorectal cancer risk
Source: Sci Rep. 2022 Nov 7;12:18852. doi: 10.1038/s41598-022-23451-y (PMC9640550; doi:10.1038/s41598-022-23451-y)
Supplement: Supplementary file 2 — Supplementary Information 2. [file 41598_2022_23451_MOESM2_ESM.docx]

Supplementary Figure 1: Flowchart for sample selection process for the data included in gene-folate interaction analyses from GECCO and CORECT.

Missing covariate/outcome data

GECCO N=12,371

CORECT N=7,017

Final included samples

GECCO N=10,748

CORECT N=16,803

Genotype data after preliminary QC

GECCO N=23,119

CORECT = 23,820

| Supplementary Table 1A. Study designs and fortification status for studies included in the analysis for predicted gene expression interaction with folate on colorectal cancer risk. | | | |
| --- | --- | --- | --- |
| **Study name** | **Study design** | **Country** | **Fortification*** |
| Alpha-Tocopherol, Beta-Carotene Cancer Prevention Study | Cohort study | Finland | No |
| Hawaii-based case-control study | Case/control | USA | No |
| Cancer Prevention Study II | Cohort study | USA | No |
| Diet, Activity and Lifestyle Study | Case/control | Germany | No |
| Health Professionals Follow-up Study | Cohort study | USA | No |
| Kentucky Cancer Registry | Case/control | USA | No |
| Melbourne Collaborative Cohort Study | Cohort study | Australia | No |
| Multiethnic Cohort Study | Cohort study | USA | No |
| Molecular Epidemiology of Colorectal Cancer | Case/control | Israel | No |
| Newfoundland Cancer Family Registry | Case/control | Canada | Yes |
| Nurses’ Health Study | Cohort study | USA | No |
| Prostate, Lung, Colorectal, & Ovarian Cancer Screening Trial | Cohort study | USA | Yes |
| Swedish Mammography Cohort and COSMs | Cohort study | Sweden | No |
| Colorectal Cancer Genetics & Genomics | Case/control | Spain | No |
| VITamin And Lifestyle Study | Cohort study | USA | Yes |
| Women’s Health Initiative | Cohort study | USA | No |
| *Studies with cases captured prior to 1998 (year of folate fortification in the USA) were considered to be non-fortified | | | |

| Supplementary Table 1B. Study design specific estimate of total folate and colorectal cancer risk, adjusting for age at reference, sex, total energy intake, and study. | | | |
| --- | --- | --- | --- |
| **Study design** | **OR for folate** | **95% Confidence Interval** | **P-value for difference in OR estimates** |
| Case/control | 0.89 | (0.86, 0.92) | 0.03 |
| Cohort study | 0.94 | (0.90, 0.98) |  |

| Supplementary Table 2A. Estimated Odds Ratio (OR) per standard deviation change in predicted gene expression within sex-study specific quantiles of total folate consumption tested for difference from the lowest quantile. | | | | |
| --- | --- | --- | --- | --- |
| Gene | **Sex-study specific quantile of total folate** | **OR** | **95% Confidence Interval** | **Interaction P-value** |
| *MTHFR* | 1 | 0.99 | 0.95, 1.07 | Ref |
|  | 2 | 1.05 | 0.97, 1.12 | 0.21 |
|  | 3 | 1.01 | 0.95, 1.08 | 0.71 |
|  | 4 | 1.02 | 0.95, 1.10 | 0.56 |

| Supplementary Table 2B. Estimated Ratio of Odds Ratio per unit change in variant weight in predicted gene expression for variants modeled in the MTFHR gene for interaction with sex-study specific quantiles of total folate consumption on colorectal cancer risk. | | | | |
| --- | --- | --- | --- | --- |
| Gene | **SNP** | **ROR Estimate** | **Standard Error** | **Interaction P-value** |
| *MTHFR* | 1:11005106_G/A | 1.000 | 0.006 | 0.999 |
|  | 1:11256916_A/G | 1.000 | 0.006 | 0.996 |
|  | 1:11721321_G/A | 1.000 | 0.006 | 0.994 |
|  | 1:11727924_C/T | 1.000 | 0.006 | 0.995 |
|  | 1:11824133_A/C | 1.000 | 0.006 | 0.997 |
|  | 1:11824575_T/C* | 1.000 | 0.006 | 0.995 |
|  | 1:11870241_T/C* | 1.000 | 0.006 | 1.000 |
|  | 1:11899033_G/T | 1.000 | 0.006 | 0.997 |
|  | 1:11932704_T/C | 1.000 | 0.006 | 0.992 |
|  | 1:11957203_G/A | 1.000 | 0.006 | 1.000 |
|  | 1:12061350_G/A | 1.000 | 0.006 | 1.000 |
|  | 1:12115691_T/C | 1.000 | 0.006 | 0.997 |
|  | 1:12230081_G/A | 1.000 | 0.006 | 0.980 |
|  | 1:12263919_C/T | 1.000 | 0.006 | 0.975 |
|  | 1:12670226_A/G | 1.000 | 0.006 | 0.998 |
|  | 1:12686912_C/T | 1.000 | 0.006 | 0.998 |
| *Variants closest to the C677T missense variant | | | | |

| Supplementary Table 2C. Estimated Ratio of Odds Ratio per additional effect allele for interaction with sex-study specific quantiles of total folate consumption on colorectal cancer risk. | | | | |
| --- | --- | --- | --- | --- |
| Gene | **SNP** | **ROR Estimate** | **95% Confidence interval** | **Interaction P-value** |
| *MTHFR* | rs1801133  (C677T) | 1.02 | 0.98, 1.06 | 0.235 |
